# Against conventional wisdom: when the public, the media, and medical practice collide

**DOI:** 10.1186/1472-6947-13-S3-S4

**Published:** 2013-12-06

**Authors:** Jakob D Jensen, Melinda Krakow, Kevin K John, Miao Liu

**Affiliations:** 1 Department of Communication, University of Utah, Salt Lake City, UT – 84112, USA

## Abstract

**Background:**

In 2009, the U.S. Preventive Services Task Force released new mammography screening guidelines that sparked a torrent of criticism. The subsequent conflict was significant and pitted the Task Force against other health organizations, advocacy groups, the media, and the public at large. We argue that this controversy was driven by the systematic removal of uncertainty from science communication. To increase comprehension and adherence, health information communicators remove caveats, limitations, and hedging so science appears simple and more certain. This streamlining process is, in many instances, initiated by researchers as they engage in dissemination of their findings, and it is facilitated by public relations professionals, journalists, public health practitioners, and others whose tasks involve using the results from research for specific purposes.

**Analysis:**

Uncertainty is removed from public communication because many communicators believe that it is difficult for people to process and/or that it is something the audience wants to avoid. Uncertainty management theory posits that people can find meaning and value in uncertainty. We define key terms relevant to uncertainty management, describe research on the processing of uncertainty, identify directions for future research, and offer recommendations for scientists, practitioners, and media professionals confronted with uncertain findings.

**Conclusions:**

Science is routinely simplified as it is prepared for public consumption. In line with the model of information overload, this practice may increase short-term adherence to recommendations at the expense of long-term message consistency and trust in science.

## Introduction

In 2009, the U.S. Preventive Services Task Force (USPSTF) announced a change in mammography guidelines, a recommendation that sparked a torrent of criticism [1-3]. Previously, the USPSTF recommended a B grade for mammography screening every 1 to 2 years for women age 40 and older. A B grade means, “The USPSTF recommends the service. There is high certainty that the net benefit is moderate or there is moderate certainty that the net benefit is moderate to substantial” [4]. In 2009, the USPSTF altered their recommendations such that biennial screening for women from age 50 to 74 received a B grade and biennial screening for women from age 40 to 49 was downgraded to a C grade. A C grade means, “Clinicians may provide this service to selected patients depending on individual circumstances. However, for most individuals without signs or symptoms there is likely to be only a small benefit from this service” [4]. The new recommendation of the USPSTF was challenged by other organizations and patient advocacy groups as it was in direct conflict with the guidelines that had been communicated—by those groups and the USPSTF—for years [2].

One interpretation of this controversy is that the USPSTF encountered problems not because their message was perceived as unsubstantiated or inaccurate, but rather that it deviated from recommendations of the past in a fairly significant manner. Past communication about mammography had focused on a simple message: Women should have an annual mammogram starting at age 40 because screening saves lives. This message was a central component of health education efforts devoted to cancer, and advocacy groups were mobilized across the United States in support of retaining annual mammographic screening as recommended practice. However, the uncertainties of the benefits and harms associated with annual mammography were rarely included in these advocacy efforts. The USPSTF had acknowledged these uncertainties in its own reports, but changing the resulting recommendation based on these uncertainties created the appearance of a discrepant message or a flip-flop [3]. Changing the recommendation for women aged 40–49 from a B grade to a C grade suggested that the USPSTF had incorrectly categorized the certainty of the mammography in the past (i.e., it went from high/moderate certainty to uncertain).

It is clear in hindsight that the USPSTF members did not fully appreciate how contradictory their recommendation was or the potential backlash it would invoke at the time that the recommendation was published [4]. The researchers and research-oriented practitioners who comprised the USPSTF believed that there were significant uncertainties concerning the value of mammography screening. Those uncertainties were known within the research community, and results from simulation research were starting to suggest the need for alterations in the screening guidelines [1]. Unfortunately, the USPSTF members and other contributors to the effort did not appreciate how the new recommendation would be received by members of the public (e.g., women at risk, community-based clinicians, public health officials) that had been steeped in messages about the value of annual screening mammography for decades. Nor did they appreciate how the public would perceive a downgrade in their recommendation.

The controversy over mammography guidelines raises significant questions about the communication of health recommendations, including: What went wrong in this situation? Who had responsibility for the significant misjudgment concerning the public reaction? How could it have been avoided? Based on these questions, we argue that this controversy is a predictable response to the systematic removal of uncertainty from the public communication of scientific content; a problem that undermines the credibility of science and confuses the public [5-10]. In other words, the 2009 mammography controversy was symptomatic of larger structural problems undermining the public dissemination of science rather than an isolated incident. The goal of this article is to articulate how conflicts of this type arise and to review possible means of redress.

## Background

The public learns about research primarily through media—television, the Internet, and newspapers. These channels are used to convey recommendations from official entities (e.g., the USPSTF), the results of single studies, and/or the accumulation of decades of research evidence [11]. This approach is necessary because few people have direct access to the research enterprise. Thus, the media disseminate research findings—a somewhat uncomfortable situation that often places media-focused enterprises and outlets at the center of scientific debates [12].

To understand the tension of this dissemination process, it is pivotal to know that scientists and journalists have distinct professional norms that often conflict [7]. Scientists value uncertainty, and this leads them to favor hedged discourse and longer, denser prose [13,14]. Journalists value concise narratives that represent myriad perspectives to achieve balanced coverage [15,16]. One can easily see this tension by comparing an academic journal article to its subsequent news coverage. Such comparisons reveal that news coverage of research often maximizes conflict by providing space to divergent voices—which are routinely manufactured or magnified to maximize conflict—and frequently omits the caveats, limitations, and uncertainties presented in the original journal reports [7,9,10,12,17-20]. For example, Lai and Lane [21] found that 43% of front-page newspaper stories about science were based on preliminary evidence. Of those stories, only 18% were described as preliminary or mentioned the limitations of the research.

Conflicting professional norms have led scientists to go through periods of media engagement and withdrawal. In the early 1900s, scientists embarked on a period of media engagement driven largely by the efforts of the Progressives—a term used to describe groups involved in a massive reform movement in the United States from approximately 1890 to 1920— who viewed research as the guiding force of reform [22]. During the Progressive Era, scientists were trained to streamline their messages when communicating with the public to ensure that science was the voice of authority in matters of policy [14,15]. This period of media engagement was followed by a significant withdrawal near the middle of the 20th century. The rationale for withdrawing was articulated best by Popper, who argued that science needed to embrace uncertainty and abandon the desire “to be right” ([23] p. 280-281). Moreover, concerns about the relationship between science and the media ultimately led others to eschew publication of research that was prematurely disseminated to the public. For example, Franz Ingelfinger, editor of the *New England Journal of Medicine* from 1967–1976, decreed in 1969 that his journal would no longer publish research that had been released to the media in advance, as “premature publicity about medical research and publicity about work that has not yet been documented cause confusion among laymen and the profession alike” ([24] p.825). Ingelfinger’s policy still allowed for interactions between scientists and media professionals, but only after research had been sufficiently vetted by the peer review process. Thus, Ingelfinger advocated a divide between scientists and media professionals to protect scientific inquiry from the negative influences of hasty public dissemination. Both Popper and Ingelfinger seemed to appreciate that media professionals value a definitive claim, and that once that claim is made it could undermine or jeopardize scientific credibility.

More recently, science seems to be moving back toward engagement. Scientists are once again seeking training in how to interact with media professionals [25,26]. Not surprisingly, this training often focuses on simplifying scientific statements so they appear more certain and (presumably) more lucid for nonscientists. The Progressives supported this approach to solidify scientists as key decision makers, but modern advocates of simplification are interested in increasing comprehension and adherence among members of larger audiences, including the general public. The health literacy and plain language movements, for example, both posit that crafting scientific information for public consumption is primarily a process of simplification [27]. The logic seems to be that many problems in the communication environment could be solved by the removal of scientific jargon and/or extraneous verbiage.

As science returns to media engagement, some have cautioned that effective communication of scientific information should be guided by the philosophy of science (e.g., Popper’s caution about avoiding the “need to be right”) and a growing body of literature evaluating the benefits and harms of removing uncertainty from scientific discourse [8,27]. The basic assumption of this approach is that conflict among scientists, the media, and the public will occur, but the emergence of such conflict should be a secondary concern among those charged with the communication of scientific information. Their primary concern should be to foster a conversation that includes uncertainty, rather than streamlining messages to achieve (what are often) short-term objectives in conveying a specific point. In the analysis section, the logic, evidence, and future directions of this alternative strategy are outlined.

## Analysis

### Streamlining and uncertainty

Two key terms need to be defined for this discussion: streamlining and uncertainty. Streamlining is the process of removing information as a message moves through communication channels. In science, Star [28] argued that streamlining often begins with researchers as they omit countless details from their research reports. Of course, streamlining is necessary as it is impossible to include all details in a message. For example, researchers might note that the temperature of their laboratory was kept at 72 F during the study but fail to mention relative humidity (as they view that as irrelevant). The streamlining process continues as research is moved forward by researchers positioning their work for publication and after publication as public relations professionals craft shorter press releases to drive media coverage. Journalists further streamline the material to fit the space requirements of their publication, and additional streamlining may occur when news coverage is reappropriated by bloggers, social media, or even in interpersonal conversation.

What information is streamlined in science communication? Two factors are systematically removed during the streamlining process: lexical complexity and uncertainty. Scientific discourse is more lexically complex than other forms of communication [29], and the removal of jargon or multisyllabic terms is standard practice when preparing a document for public consumption [27]. This practice often lowers the reading level of the message, which may benefit audiences with lower literacy [30], although the costs and benefits of reducing lexical complexity have yet to be fully investigated [27].

In an effort to reduce lexical complexity, communicators frequently cut uncertainty from the message as well. Uncertainty is both a perception and a message feature. A person can feel uncertain and a message can convey uncertainty. Brashers argued that uncertainty is a complex self-perception that a situation is “ambiguous, complex, unpredictable, or probabilistic,” and it occurs “when information is unavailable or inconsistent” ([31] p.478). As a message feature, uncertainty is cut to reduce lexical complexity and because many communicators believe that audiences want to reduce or avoid uncertainty [32-34]. Uncertainty management theory, on the other hand, posits that people sometimes prefer uncertainty [31]. Identifying when and why people prefer uncertainty is the primary objective of uncertainty management research.

Uncertainty in science comes in at least two forms: lexical and discourse-based [35]. Lexical uncertainty occurs when a communicator uses hedging (e.g., may, if, perhaps, might) to suggest uncertainty. “Blueberries may prevent throat cancer” is a claim with lexical uncertainty whereas “blueberries prevent throat cancer” does not contain lexical uncertainty. Discourse-based uncertainty occurs when a communicator provides a reason that a claim is uncertain. For instance, if researchers note that a study used tomato powder instead of tomatoes, and thus the impact of tomatoes is still unknown, that would be an example of discourse-based uncertainty.

## Streamlining, uncertainty, and the public communication of science

Public communication of science moves fast, perhaps faster than scientists recognize. The desire for definitive information on pressing issues of the day can foster a culture of short, overly certain messages that seem to change over time. An infamous example of this tendency is news coverage of margarine and butter. For several decades, researchers have examined the relative health benefits of margarine and butter. Individual studies have yielded data supporting one or the other (and sometimes neither), which has led to a series of stories touting margarine over butter, then butter over margarine, then margarine over butter, and so on [36,37]. Research on this topic is relatively uncertain, yet news coverage has often presented the issue as certain and in line with the findings of each new study. The margarine-versus-butter storyline is typical of news coverage in that science is often presented in brief stories that seem to flip-flop over time [38]. This flip-flopping is driven by journalistic norms that cut lexical and discourse-based uncertainty and favor conflict and newsworthiness [39].

Despite a renewed interest in simplification, the reality is that public communication of science typically is simple (in the short term), and this fact can produce confusion (in the long term). For example, the controversy about the USPSTF mammography guidelines was predicated by decades of simple, adherence-focused communication. Simple messages advocating annual mammography increased adherence among U.S. women over 40 years of age from 29% in 1987 to 70.4% in 2000 [40]. Streamlining communication may maximize behavioral response, but that same simplicity potentially triggers backlash if the recommendation needs to be changed. In other words, the controversy about the USPSTF mammography guidelines was, in many respects, a classic margarine-versus-butter situation.

## Uncertainty and the public

Research suggests that many adults have limited health and science literacy [27]. In light of these skill deficiencies, communicating scientific uncertainty to the public may sound like a misguided idea. However, lexical complexity and uncertainty are distinct message features. That is, there is nothing about uncertainty that requires lexical complexity. For example, the following sentences are written at a first-grade reading level (Flesch-Kincaid Grade Level = 0.6), contain no passive sentences, and have a reading ease score of 100%: “This study used mice. Mice and people are not the same. We do not know if it will work in people.” In terms of science literacy, existing measures of this construct do not test comprehension of scientific uncertainty; therefore, the public’s ability to process uncertain scientific statements is still largely unknown [34].

What is known is that, among the public, there is widespread fatalism and overload concerning health information. The Health Information National Trends Survey (HINTS) is a national survey of U.S. adults conducted approximately every other year. Focused primarily on cancer, the HINTS data have shown that: 27% of adults believe, “There's not much people can do to lower their chances of getting cancer”; 41% agree with the statement, “It seems like almost everything causes cancer”; and 71.5% express the view that, “There are so many recommendations about preventing cancer, it's hard to know which ones to follow” [41]. The first two beliefs are examples of cancer fatalism, whereas the last belief is cancer information overload [42]. Adults who embrace ideas associated with higher fatalism and overload are less likely to engage in cancer prevention and detection behaviors [42,43].

Importantly, fatalism and information overload are negatively correlated with education [42,44]. That is, people with less education are more likely to exhibit fatalism and overload. This relationship suggests that education provides something that allows adults to process cancer information without triggering negative reactions. It is possible that education provides the basic literacy and numeracy skills necessary to comprehend news coverage of research (news coverage is typically written at a ninth-grade reading level [45]). It is also possible that education provides a context for understanding news coverage. Research to date has shown that including discourse-based uncertainty in news coverage of cancer research decreases cancer fatalism and nutritional backlash [8]. No study has examined whether uncertainty is related to information overload.

## The future of uncertainty

The first question that needs to be addressed is whether lay adults can meaningfully process scientific uncertainty. Studies examining the positive and negative effects of textual and discourse-based uncertainty would be especially useful. Uncertainty management theory posits that people respond to uncertain information in complex ways (e.g., avoidance, engagement, anger, relief, confusion). How people respond to uncertainty will likely depend on the type of uncertainty, how it is communicated, and individual skill and dispositions [34,46]. From a theoretical standpoint, the controversy about the USPSTF mammography guidelines raises questions about the concealment of uncertainty. Is uncertainty perceived differently if people believe it was initially downplayed or omitted from discourse? The perception that information was withheld may have detrimental impact on the credibility of the communicator, as well as identity issues for those who advocated the original message. Indeed, the mammography controversy frustrated many screening advocates who had personally endorsed a course of action (and message) that was now being questioned. Understandably, many screening advocates felt betrayed, embarrassed, and angry. All of these reactions raise questions about logic and timing in communicating uncertainty. Of course, only the aggregation of numerous studies across different contexts, time intervals, and forms of uncertainty will yield generalizable knowledge to guide effective communication practice [7,8,33,34].

The USPSTF was hindered by a suboptimal communication strategy about the mammography controversy, but they may be working in the right direction in a larger sense. For example, one USPSTF goal is to categorize the state of knowledge on particular public health issues. Part of this categorization process is an assessment of the level of certainty regarding the net benefit of a health behavior. Evidence is categorized as low, moderate, or high in terms of certainty (see Table 1 for the criteria of each category [47]). Categorizing certainty is a potentially useful idea, and research should investigate public comprehension of the categories. However, health researchers should also consider whether the number of categories—three at present—is sufficient. The goal is to provide a sufficiently nuanced categorization scheme to allow researchers to accurately describe the evolution of a research program. It could be argued that the categorization scheme currently used by the USPSTF failed the 2009 Task Force, as their desired course of action (i.e., a change in the recommendation based on growing uncertainties) could not be properly conveyed. An alternative categorization scheme could focus on the state of the research with categories like no studies, isolated, infancy, emerging, and established. The model of information overload posits that people need to categorize information to process scientific research meaningfully [8]; therefore, cultivating a widely recognized and sufficiently detailed categorization scheme could be a valuable addition to public communication of science.

**Table 1:** How the U.S. Preventive Services Task Force categorizes level of certainty

Level of Certainty	Description
High	The available evidence usually includes consistent results from well-designed, well-conducted studies in representative primary care populations. These studies assess the effects of the preventive service on health outcomes. This conclusion is therefore unlikely to be strongly affected by the results of future studies.
Moderate	The available evidence is sufficient to determine the effects of the preventive service on health outcomes, but confidence in the estimate is constrained by such factors as: • the number, size, or quality of individual studies • inconsistency of findings across individual studies • limited generalizability of findings to routine primary care practice • lack of coherence in the chain of evidence As more information becomes available, the magnitude or direction of the observed effect could change, and this change may be large enough to alter the conclusion.
Low	The available evidence is insufficient to assess effects on health outcomes. Evidence is insufficient because of: • the limited number or size of studies • important flaws in study design or methods • inconsistency of findings across individual studies • gaps in the chain of evidence • findings that are not generalizable to routine primary care practice • a lack of information on important health outcomes More information may allow an estimation of effects on health outcomes.

Note: Evidence regarding the net benefit of health behaviors is categorized as low, moderate, or high using the above criteria.

One intriguing area of future research is the study of visual depictions of uncertainty [48]. Effective visuals may overcome literacy and numeracy deficits. Visuals could also convey complexity more efficiently and address space issues that often drive the streamlining process. The 2009 mammography controversy may have been avoided if communicators had effective visuals formats for contextualizing the magnitude of uncertainty about mammography, especially as that research unfolded over time. Concerning the latter, a timeline visual depicts key milestones/events in a way that establishes the duration of activities (years, decades, centuries), evolution of ideas, and level of uncertainty. In practice, timeline visuals should encourage both communicators and audiences to consider the totality of a situation rather than focusing on the details of the latest event (only). Timelines have only been in use for approximately 250 years, and the public initially struggled to comprehend this new visual format [49]. Social scientific research on the use and efficacy of this visual format remains in its infancy. There is evidence that timelines enhance information recall [50], but social scientific research concerning comprehension is limited. This is an interesting omission in the research given the tendency of visual researchers to use timeline-oriented visuals (e.g., Florence Nightingale’s visual depiction of deaths during the Crimean War [1853–1856]) as exemplars of quality [48]. Visuals may also be ideal to communicate context for the magnitude of the uncertainty (henceforth, context-magnitude visuals). Researchers often use context-magnitude visuals to demonstrate the relative size of an effect; for example, scholars have contextualized the relationship between exposure to media violence and aggression by visual depicting other (weaker, stronger, comparable) relationships at the same time [51]. Similar visuals could be constructed and evaluated for communicating the magnitude (or even form) of scientific uncertainty in a given situation. Of course, researchers should also be cautious because there is a risk that communicating uncertainty in this fashion could mislead or confuse target audiences.

Another promising area for future research is the communication of uncertainty via interactive media. Media are evolving in ways that challenge traditional journalism practices including the organization and form of content. Interactivity allows content to unfold at the discretion of the consumer, and this provides a vehicle for conveying complex information to diverse audiences in meaningful ways [27].

## Conclusions

Competition between conflicting opinions is a healthy part of public discussion in situations defined by uncertainty. Silencing dissenting opinions when an optimal course of action is unclear creates a potentially hostile communication environment. That aside, conflict that derives from known biases in communication channels is a concern. Such conflict may stem from systematic efforts to simplify information for public consumption. Though well intentioned, simplification strategies may have unintentional negative impacts on certain individuals or population subsets such as the cultivation of fatalism, backlash, and overload [8]. Simplification strategies could also undermine or damage the credibility of the science [7].

Practitioners and media professionals will be interested in possible solutions. Continued research of uncertainty management, uncertainty categorization, and visual depictions of uncertainty may identify promising communication strategies for specific populations and situations. Until then, communicators should consider the long-term goals/consequences of their strategies in addition to the philosophical and ethical foundations of science. Even if future research suggests that adults with skill deficits struggle to process uncertainty, and that this contributes to problems they have in managing their own health care, communicators will be faced with the challenge of determining if it is ethical to conceal such information from populations with literacy deficits or the population as a whole [34]. Cutting, removing, or simplifying information for public consumption is (once again) a popular strategy. Yet, simplification, in and of itself, it is not a virtue of communication, even if it may be effective at achieving some goals. Simplification is a message strategy or feature that can yield positive and negative effects. Rather than focusing solely on simplification as a goal, communicators should strive to be meaningful and to embrace strategies that achieve that goal regardless of their simplicity or complexity. Meaningful health recommendations may need to include indicators of uncertainty even if doing so sacrifices short-term adherence for long-term coherence.

## List of abbreviations used

HINTS: Health Information National Trends Survey; USPSTF: U.S. Preventive Services Task Force

## Competing interests

The authors have no competing interests to declare.

## Authors' contributions

JDJ, MK, KKJ, and ML helped to draft and revise the manuscript. All authors read and approved the final manuscript.

## Declarations and disclaimer

The Eisenberg Conference Series 2012, *Supporting Informed Decision Making When Clinical Evidence and Conventional Wisdom Collide*, was conducted in Rockville, Maryland, by the John M. Eisenberg Center for Clinical Decisions and Communications Science, Baylor College of Medicine, Houston, Texas, for the Agency for Healthcare Research and Quality under Contract No. HHSA 290-2008-10015-C. Publication costs for this supplement were funded by this contract. The author of this article is responsible for its content. No statement may be construed as the official position of the Agency for Healthcare Research and Quality and of the U.S. Department of Health and Human Services.

This article has been published as part of *BMC Medical Informatics and Decision Making* Volume 13 Supplement 3, 2013: Articles from the Eisenberg Center Conference Series 2012: Supporting informed decision making when clinical evidence and conventional wisdom collide. The full contents of the supplement are available online at http://www.biomedcentral.com/bmcmedinformdecismak/supplements/13/S3.
